# Impact of Gender on Insomnia

**DOI:** 10.3390/brainsci13030480

**Published:** 2023-03-12

**Authors:** Jolijn Boer, Nadya Höhle, Lisa Rosenblum, Ingo Fietze

**Affiliations:** Department of Internal Medicine and Dermatology, Interdisciplinary Center of Sleep Medicine, Charité—Universitätsmedizin Berlin, 10117 Berlin, Germany

**Keywords:** insomnia, gender, sleep, sleep disorder, phenotypes, gender-specific treatment

## Abstract

There is a distinct preponderance of female insomniacs when compared to male insomniacs. The aim of this study was to examine possible gender differences in the causes for insomnia, and the phenotypes of insomnia, and to investigate whether gender-specific insomnia diagnosis and treatment could be relevant in clinical practice. Data were collected from 121 insomniac patients by a medical specialist in the framework of normal clinical practice in Germany. The data consist of the patient’s medical history and various sleep-related patient questionnaires. Data from both genders were tested for independence using chi-square tests and Mann–Whitney U tests. We found a correlation between the gender of the patient and insomnia phenotypes in several aspects: concomitant lipometabolic disorders, diabetes mellitus, and high BMIs are more common in male insomniacs (*p* < 0.05). Frequency of insomnia occurrence in certain age groups, insomnia severity, distribution of SOI (sleep onset insomnia), SMI (sleep maintenance insomnia) and combined SOI + SMI, sleep duration, the time needed to seek medical consultation, trying out sleep-inducing drugs/techniques and the trigger, etiology and familial predisposition of the insomniac disorder were independent of the patient’s gender. We would like to re-evaluate the results with a larger number of patients in a further study.

## 1. Introduction

In healthcare, gender-specific medicine considers both physiological and biological variances (sex differences) as well as sociocultural disparities (gender differences) regarding pathogenesis, diagnosis, and treatment in healthcare [1,2,3]. Gender also has an influence on health patterns, psychosocial patterns, sociodynamics patterns, and access to healthcare [3]. The specialized approach of gender-specific medicine is a possibility to overcome these barriers and increase therapy adherence and therapy success. One example could be to implement female-only treatment settings to provide a secure surrounding for females and to focus on female-specific issues, such as co-occurring disorders, family responsibilities, and parenting. These could also offer childcare services for women with children who cannot find time for treatment [4,5,6]. Gender-specific treatment services are already common for patients with substance abuse disorders [7]. However, they are not so common for other diseases, such as type 2 diabetes, and here experts see the opportunity for gender-specific treatment to increase quality of life and survival [8]. Just like for type 2 diabetes, there is currently no gender-specific treatment for insomnia as of yet.

The prevalence of insomnia is high and varies greatly [9,10,11]. Remission rates for chronic insomnia are low [12]. Online surveys conducted between May and September 2020 suggest that the prevalence increased during and due to the COVID-19 pandemic: In Europe, 8.2% to 25.6% of all respondents were found to have a probable insomnia disorder [13,14]. This increase could already be detected at the beginning of the COVID-19 pandemic in February 2020, especially among females [15]. However, not only did the COVID-19 pandemic have an impact on prevalence, it also affected the management of insomnia [16].

Insomnia increases the risk of heart attacks, heart failure, high blood pressure, anxiety disorders, and suicide, just to name a few [17,18,19,20,21,22,23,24]. Consequently, this sleep disorder results in high direct and indirect, social and economic costs, especially when left untreated [25]. This includes healthcare utilization, deadweight loss from inefficiencies in taxation/social assistance, medication, therapy, sick leave, and early retirement [25,26]. A study from 2010 found that sleep disorders, including insomnia, narcolepsy, sleep apnea, and hypersomnia, resulted in costs of around EUR 790 per patient a year in Europe [27].

Phenotyping insomnia patients in general is still under development. So far, common classification criteria for insomnia include total sleep duration, insomnia severity, and psychological stress [28,29]. A total sleep duration of <6 h identifies more pronounced insomnia, increased psychological impairment, and a possible genetic predisposition. This subtype may benefit less from cognitive behavioral therapy than insomnia patients with longer sleep duration, where the cause is less likely to be genetic and more likely to be psychological [28]. Insomnia severity can be well assessed with the validated Insomnia Severity Index (ISI) questionnaire, which allows the classification of insomnia into mild, moderate, and severe [30]. Daytime sleep, stability of insomnia, and better sleep in unfamiliar environments are also interesting for the classification of insomnia. However, the clinical benefit of these classifications has yet to be identified [31].

It is known that there is a distinct preponderance of female insomniacs when compared to male insomniacs [32]. A possible explanation for this finding can be based on differences in sex hormones [33,34,35]. Another reasonable explanation is the higher prevalence of depression and anxiety disorders in women [36,37,38], which are high-risk factors for insomnia [39,40,41]. A study on Chinese insomniacs found that unemployment is correlated to insomnia in men and marital status is correlated to insomniac disorders in women [42]. Another Chinese cohort of 9851 subjects indicated that being divorced or widowed was only correlated to insomnia in women [39]. A cross-sectional study in Taiwan suggests that no or irregular exercise, using sleeping pills, and suffering from Restless Legs Syndrome were correlated to insomnia in males. Poor appetite has been shown to be relevant in female insomniacs [43].

For the first time, we will examine differences in the causes for insomnia and the phenotypes of insomnia when comparing genders in a German population. Additionally, we will examine whether gender-specific insomnia diagnosis and treatment could be relevant in clinical practice.

## 2. Materials and Methods

### 2.1. Participants and Recruitment

We collected data from 121 patients who were already diagnosed with insomnia (difficulties initiating sleep, maintaining sleep, or early morning awakening) according to the ICSD-3 criteria. Recruitment took place at the Interdisciplinary Center of Sleep Medicine at Charité—University Medicine Berlin, Germany. Patients over the age of 18 were included in the study. Another requirement for participation was a signed informed consent on the use of their pseudonymized data for research purposes. Patients with paradoxical insomnia were excluded from the study. The study was conducted in compliance with the declaration of Helsinki, and the study was approved by the Ethics Committee of the Charité—University Medicine Berlin, Germany (Research Ethics Committee Reference Number: EA4/204/22).

### 2.2. Procedure

The data were collected by a medical specialist in the framework of normal clinical practice after the insomnia diagnosis was confirmed. This consisted of collecting the medical history of the patients as well as various self-report questionnaires: Insomnia Severity Index (ISI), Beck Depression Inventory (BDI-II), Epworth Sleepiness Scale (ESS), Restless Legs Syndrome-Diagnostic Index (RLS-DI) and STOP-BANG questionnaire. Categorized results from the ISI questionnaire were used for the analysis: 0–7 points = normal finding; 8–14 points = mild insomnia; 15–21 points = moderate insomnia and >22 points = severe insomnia [30]. The BDI-II scores were also categorized into groups for the analysis: minimal (BDI-score 0–13), mild (BDI-score 14–19), moderate (BDI-score 20–28), and severe depression (BDI-score 29–63) [44]. All data were pseudonymized and entered into a database (Excel Version 16.70 and SPSS Version 28.0.1.0).

### 2.3. Statistics

Statistical analysis was carried out in SPSS (IBM SPSS Statistics 28.0.1.0). We used chi-square tests to test the independence of two variables, with Cramer’s V being the measure of the effect size of the test result. Parametric unpaired *t*-tests were used to examine whether the means of two independent measurement groups differed from one another and the Mann–Whitney U test was used as a non-parametric alternative to the *t*-test if the criteria of normal distribution were not met by the data. In cases where individual questions were omitted, we excluded these patients from the analysis of the according question and adjusted the number of patients to the variable. This leads to the varying number of patients “N” in the evaluation.

## 3. Results

### 3.1. Patient Characteristics

Out of the 121 patients in our study cohort, 58.7% (*n* = 71) were female. The average age was 54.7 ± 15.1 years (range: 21–85). Nine patients were under 30 years old. More than half of the patients (64.5%) had a combined SOI (sleep onset insomnia) and SMI (sleep maintenance insomnia) and had been suffering from insomnia for 9.6 ± 9.9 years until they consulted a sleep specialist. One-third (33.6%) of the study population was concomitantly suffering from a type of cardiovascular disease, including arterial hypertension, cardiac arrhythmia, coronary artery disease, or myocardial infarction. All collected patient characteristics are shown in Table 1.

### 3.2. Correlation with Gender

#### 3.2.1. Age

There was no significant correlation between the occurrence of insomnia in certain age groups (21–40 years, 41–60 years, >60 years) and the gender of the patient (χ^2^ (2) = 2.77, *p* = 0.25, V = 0.15).

#### 3.2.2. Sleep Parameters

A chi-square test for independence was performed to investigate the relationship between gender and the insomnia severity. There was no statistically significant connection between the mentioned parameters (χ^2^ (3) = 3.27, *p* = 0.35, V = 0.17). The chi-square test was repeated with merged subgroups (no or mild insomnia versus moderate to severe insomnia) to eliminate the interference factor of too low cell frequencies. However, the final result was still not significant: χ^2^ (1) = 0.27, *p* = 0.61, V = 0.05.

**Figure 1:**
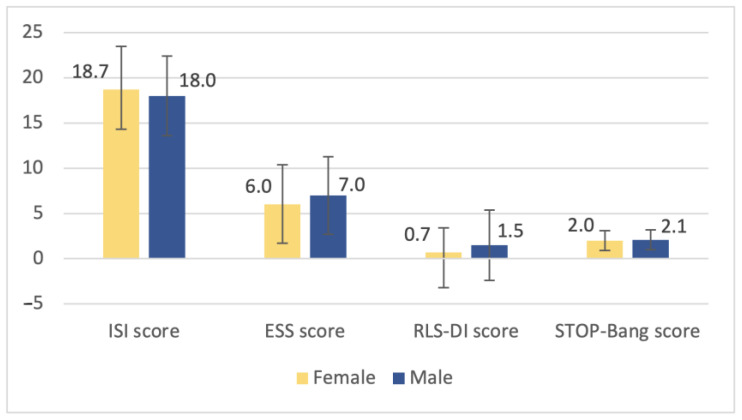
Results of the sleep-related patient questionnaires in points. ISI = Insomnia Severity Index, ESS = Epworth Sleepiness Scale, RLS-DI = Restless Legs Syndrome-Diagnostic Index.

The ISI score was compared between males and females and showed no significant difference (mean ISI score female: 18.7; mean ISI score man: 18.0; unpaired *t*-test t (112) = 0.78, *p* = 0.44) (see Figure 1).

**Figure 2:**
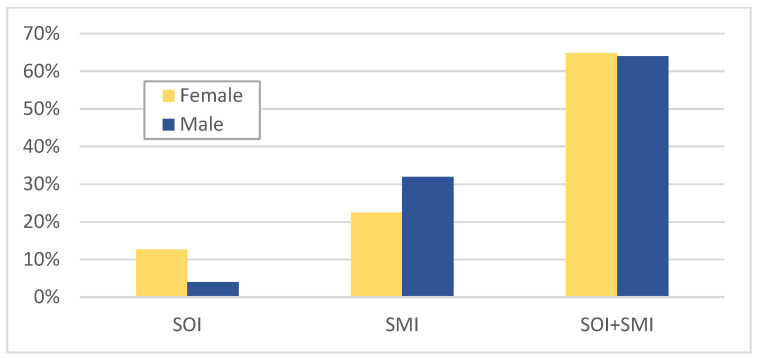
Distribution of SOI (sleep onset insomnia), SMI (sleep maintenance insomnia) and SOI + SMI (combined sleep onset insomnia and sleep maintenance insomnia) between genders.

While more women were affected by SOI and more men by SMI, combined SOI and SMI was equally common in both genders (see Figure 2). A chi-square test between the variables indicates no statistical significance: χ^2^ (2) = 3.43, *p* = 0.18, V = 0.17.

There was also no significant difference in sleep duration (during the week) between genders (Mann–Whitney U test: U = 1436.50, Z = 0.72, *p* = 0.47).

#### 3.2.3. Time Needed to Seek Medical Consultation

On average it took the patients 9.6 ± 9.9 years to seek medical consultation. The difference between genders was analyzed with a Mann–Whitney U test. The test was not significant: U = 1286.50, Z = −1.22, *p* = 0.22.

#### 3.2.4. Previous Sleep-Inducing Drugs/Techniques

**Figure 3:**
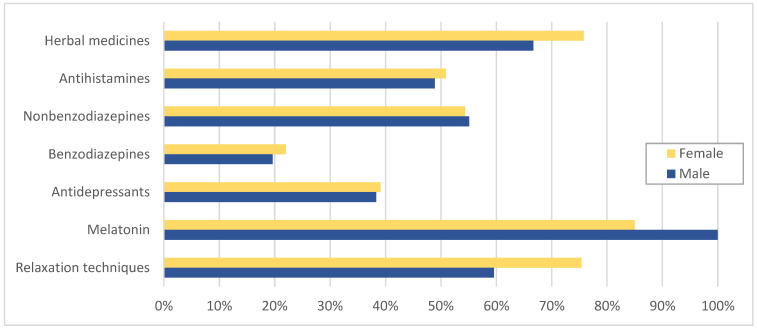
Previously tested sleep-inducing drugs/techniques.

There was no significant difference between genders in treatment attempts, that had been made before the patients came to the sleep medicine outpatient clinic (see Figure 3).

The individual results of the chi-square tests with the respective dependent variables are displayed in Table 2.

#### 3.2.5. Preexisting Conditions

**Figure 4:**
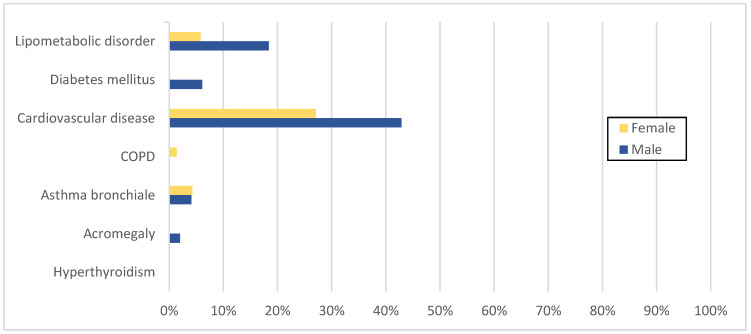
Concomitant diseases of insomnia patients.

Multiple chi-square tests were calculated to examine the gender-specific distribution of concomitant diseases (see Table 3). The test shows a significant correlation between the gender of the patient and the frequency of occurrence of diabetes mellitus (type 2) and lipometabolic disorder in insomnia patients (χ^2^ (1) = 4.34, *p* = 0.04, V= 0.20 and χ^2^ (1) = 4.62, *p* = 0.03, V = 0.20, respectively). Lipometabolic disorders and diabetes mellitus are more common in men suffering from insomnia (see Figure 4). An unpaired *t*-test for the variables gender and BMI was carried out with regard to the more frequent occurrence of lipometabolic disorders in the male insomnia population. This became significant with a *p*-value < 0.05: t (114) = −2.87, *p* = 0.01.

Patients were grouped into different categories according to their BDI scores (minimal = BDI score 0–13, mild = BDI score 14–19, moderate = BDI score 20–28 and severe depression = BDI score 29–63) in order to investigate whether concomitantly suffering from depression is more common in female insomniacs. For the comparison of frequencies of minimal, mild, moderate and severe depression between genders, the chi-square test indicated no statistical significance i.e., correlation: χ^2^(1) = 0.75, *p* = 0.39.

#### 3.2.6. Etiology of the Insomniac Disorder

The known triggers of the insomniac disorder in males and females did not differ significantly (χ^2^(1) = 0.00, *p* = 0.98). A possible correlation between gender and an idiopathic etiology of insomnia was examined using a chi-square test. This also showed no significant result: χ^2^ (1) = 1.41, *p* = 0.24. A total of 44.2% of the study population reported a familial predisposition to sleep disorders. A chi-square test showed no statistical significance between males and females (χ^2^(1) = 0.09, *p* = 0.76).

## 4. Discussion

In this study on insomnia, patients were predominantly female (59.0% vs. 41.0%), mirroring the results of previous studies [31,32,45,46]. The majority of patients (81.6%) were suffering from moderate insomnia (ISI score: 15–21 points) [30]. The mean BDI-II, ESS, STOP-Bang, and RLS-DI scores of 12.7 points, 6.4 points, 2 points, and 1 point can be translated into no indication for depression, slightly increased daytime sleepiness, low risk for obstructive sleep apnea, and a mild restless legs syndrome [47,48,49,50].

No statistically relevant connection could be made between the frequency of occurrence of insomnia in certain age groups when comparing genders. In contrast to our results, a study from France examining elderly insomniacs shows that women tend to be younger [51]. On the other hand, a registry of 2144 adults in Spain found there was a steady deterioration of the sleep quality in women with increasing age, whereas this worsening was not as constant in men. The age range of this registry was, however, somewhat higher and narrower than in our study (43–71 years vs. 21–85 years) [52].

The insomnia severity also seemed to not be related to gender (ISI score 18.0 ± 4.8 in males vs. 18.7 ± 4.4 in females, *p* = 0.44). This is consistent with a cross-sectional study in Korea, where the ISI score comparing 260 male and female insomniacs showed no statistically significant difference (14.0 ± 4.3 vs. 14.6 ± 4.4, *p* = 0.199) [53].

The influence of gender on subjective sleep duration was not significant in our study. This finding is supported by the results of the Spanish registry, where this correlation also showed no significance [52].

In our study, the distribution of SOL, SMI, and combined SOI + SMI also seems to be independent of the gender of the patient. This result is consistent with a study of 260 Korean insomniacs [53]. It does not, however, comply with the results of an online survey conducted in Norway, a study from France, or a Taiwanese cross-sectional study, which found that SOL alone was more common in women than in men [43,45,51].

Our results show that gender is also not related to how long insomnia symptoms persist or which drug treatment attempts are made before a sleep specialist is seen. On average it took 9.6 ± 9.9 years until our patients went to see a sleep specialist and most (72.0%) had previously tried herbal medicines. A study on French elderly insomniacs shows that women tend to use sleep-inducing drugs (including benzodiazepines, nonbenzodiazepines, antihistamines, and herbal medicines) slightly more often than men [51].

Around 40% of our study population could name a trigger for their insomniac disorder, the most common reasons being psychological and/or familial (separation or death of a loved one, stress, and children). Even though females tend to suffer more from stressful life events than men [54], our study showed that the trigger of the insomniac disorder is independent of gender. The hypothetical influence of gender on the etiology and familial predisposition of insomnia can also be rejected from our analysis.

Our findings suggest that there is no significant connection between insomnia comorbid with depression and the gender of the patient. This contradicts the expectation, in that depression is more common in females in general [36,37,55,56,57]. On average, the BMI of the total population was within the normal weight range of 24 ± 5 kg/m^2^ [58]. In our study, diabetes mellitus (*p* = 0.04), lipometabolic disorders (*p* = 0.03), and obesity (higher BMI) (*p* = 0.01) as concomitant diseases of insomnia are more common in men. A French study of elderly insomniacs supports the statement that men with insomnia tend to have a higher BMI and, in contrast to our findings, it shows that women are more likely to concomitantly suffer from depression [51].

The aim of the study was to examine whether there are differences in the phenotype of insomnia when comparing genders. The literature shows contradictory results. We would like to encourage scientists to explore this hypothesis further to complement the phenotyping of insomnia and, within this framework, personalize treatment and possibly establish gender-specific insomnia diagnostics and treatment.

### Limitations

The study was intended to discover what impact gender has on insomnia. Only very few aspects were found showing a correlation between the gender of the patient and insomnia phenotypes. However, it is important to note that even though many possible factors were analyzed (reaching from triggers for insomnia, sleep duration, and insomnia severity to the family predisposition) many other factors such as alcohol, drug use, nightmare recall, menopause, employment, social conditions, and children were not taken into account. It is also important to consider differences in sleep homeostasis and circadian rhythms between the genders. For example, studies suggest that women have higher basal sleep pressures [59,60] and that their intrinsic circadian period is significantly shorter [61]. This could play an important role in the development of personalized treatment for insomniacs.

Another limitation of the study is the rather small sample size. As a result, the frequencies of the chi-square test were often too low, making the interpretation of the result less reliable. In addition, certain questions were often left out by the patients, e.g., questions regarding depression as a pre-existing condition.

## 5. Conclusions

Overall, our cohort showed that the gender of the patient only has a minor influence on the phenotype of insomnia. We are currently continuing our data collection and would like to re-evaluate the results with a larger number of patients. Further studies should also investigate the role of other moderators such as age, origin, fitness, nutrition, medical comorbidities, and medications to better understand the pathophysiology and thus the phenotype of insomnia.

## Figures and Tables

**Table 1:** Sample description.

	Total *n* (%), Unless Stated Otherwise	Female *n* (%), Unless Stated Otherwise	Male *n* (%), Unless Stated Otherwise
Age ^a^			
Mean ± SD [years]	54.7 ± 15.1	55.5 ± 14.1	53.4 ± 16.5
21–40	24 (19.8%)	11 (15.5%)	13 (26.0%)
41–60	50 (41.3%)	33 (46.5%)	17 (34.0%)
0	47 (38.9%)	27 (38.0%)	20 (40.0%)
Current sleep disorder ^a^			
SOI	11 (9.1%)	9 (12.7%)	2 (4.0%)
SMI	32 (26.4%)	16 (22.5%)	16 (32.0%)
SOI + SMI	78 (64.5%)	46 (64.8%)	32 (64.0%)
ISI score ^b^			
Mean ± SD [points]	18.4 ± 4.6	18.7 ± 4.8	18.0 ± 4.4
0–7	1 (0.9%)	1 (1.5%)	0 (0.0%)
8–14	22 (19.3%)	14 (20.3%)	8 (17.8%)
15–21	63 (55.3%)	34 (49.3%)	29 (64.4%)
22–28	28 (24.6%)	20 (29.0%)	8 (17.8%)
BDI-II score ^c^			
Mean ± SD [points]	12.7 ± 9.2	13.0 ± 9.9	12.3 ± 8.2
Sleep parameters			
Mean sleep duration ± SD ^d^ [h]	5.0 ± 1.6	5.1 ± 1.5	4.8 ± 1.7
Mean ESS score ± SD ^e^ [points]	6.4 ± 4.4	6.0 ± 4.4	7.0 ± 4.3
Mean RLS-DI score ± SD ^f^ [points]	1.0 ± 3.2	0.7 ± 2.7	1.5 ± 3.9
Mean STOP-Bang score ± SD ^g^ [points]	2.0 ± 1.1	2.0 ± 1.1	2.1 ± 1.1
BMI ^h^			
Mean ± SD [kg/m^2^]	24.1 ± 4.7	23.1 ± 4.6	25.5 ± 4.4
<18.5 kg/m^2^	6 (5.2%)	4 (5.7%)	2 (4.3%)
18.5–25 kg/m^2^	73 (62.9%)	51 (72.9%)	22 (47.8%)
25–30 kg/m^2^	23 (19.8%)	9 (12.9%)	14 (30.4%)
30–35 kg/m^2^	10 (8.6%)	5 (7.1%)	5 (10.9%)
35–40 kg/m^2^	3 (2.6%)	0 (0.0%)	3 (6.5%)
>40 kg/m^2^	1 (0.9%)	1 (1.4%)	0 (0.0%)
Pre-existing conditions			
Lipometabolic disorder ^i^	13 (11.0%)	4 (5.8%)	9 (18.4%)
Diabetes mellitus ^i^	3 (2.5%)	0 (0.0%)	3 (6.1%)
Cardiovascular disease ^j^	40 (33.6%)	19 (27.1%)	21 (42.9%)
COPD ^i^	1 (0.8%)	1 (1.4%)	0 (0.0%)
Asthma bronchiale ^i^	5 (4.2%)	3 (4.3%)	2 (4.1%)
Acromegaly ^k^	1 (0.9%)	0 (0.0%)	1 (2.0%)
Hyperthyroidism ^l^	0 (0.0%)	0 (0.0%)	0 (0.0%)
Previous sleep-inducing drugs/techniques			
Herbal medicines ^m^	79 (71.8%)	47 (75.8%)	32 (66.7%)
Antihistamines ^n^	52 (50.0%)	29 (50.9%)	23 (48.9%)
Nonbenzodiazepines ^o^	64 (54.7%)	37 (54.4%)	27 (55.1%)
Benzodiazepines ^p^	22 (21.0%)	13 (22.0%)	9 (19.6%)
Antidepressants ^q^	43 (38.7%)	25 (39.1%)	18 (38.3%)
Melatonin ^r^	29 (90.6%)	17 (85.0%)	12 (100.0%)
Relaxation techniques ^s^	80 (69.0%)	52 (75.4%)	28 (59.6%)
Trigger of the insomniac disorder ^t^			
Separation from loved one	7 (8.4%)	3 (5.8%)	4 (12.9%)
Stress	6 (7.2%)	3 (5.8%)	3 (9.7%)
Death of a family member	5 (6.0%)	4 (7.7%)	1 (3.2%)
Children	6 (7.2%)	5 (9.6%)	1 (3.2%)
Onset of menopause	5 (6.0%)	5 (9.6%)	0 (0.0%)
Medication	2 (2.4%)	2 (3.8%)	0 (0.0%)
Jet lag	2 (2.4%)	1 (1.9%)	1 (3.2%)
Other	19 (22.9%)	10 (19.2%)	9 (29.0%)
None	34 (41.0%)	20 (38.5%)	14 (45.2%)
Etiology of the insomniac disorder			
Familial predisposition to sleep disorders ^u^	50 (44.2%)	30 (45.5%)	20 (42.6%)
Idiopathic etiology ^f^	65 (59.1%)	42 (63.6%)	23 (52.3%)

Data available from the following number of patients: ^a^ N = 121 (71 + 50); ^b^ N = 114 (69 + 45); ^c^ N = 109 (64 + 45); ^d^ N = 113 (65 + 48); ^e^ N = 118 (70 + 48); ^f^ N = 110 (66 + 44); ^g^ N = 98 (55 + 43); ^h^ N = 116 (70 + 46); ^i^ N = 118 (69 + 49); ^j^ N = 119 (70 + 49); ^k^ N = 111 (66 + 45); ^l^ N = 100 (55 + 45); ^m^ N = 110 (62 + 48); ^n^ N = 104 (57 + 47); ^o^ N = 117 (68 + 49); ^p^ N = 105 (59 + 46); ^q^ N = 111 (64 + 47); ^r^ N = 32 (20 + 12); ^s^ N = 116 (69 + 47);^t^ N = 83 (52 + 31); ^u^ N = 113 (66 + 47). SOI: sleep onset insomnia, SMI: sleep maintenance insomnia, BDI-II: Beck Depression Inventory, ESS: Epworth Sleepiness Scale, RLS-DI: Restless Legs Syndrome-Diagnostic Index, SD: standard deviation, n: number of patients, N: number of patients (number of female patients + number of male patients).

**Table 2:** Correlation between gender and previously tested sleep-inducing drugs. Results of the chi-square tests with a significance level of 0.05.

	χ^2^ (1)	*p*
Herbal medicines	1.116	0.29
Antihistamines	0.039	0.84
Nonbenzodiazepines	0.006	0.94
Benzodiazepines	0.095	0.76
Antidepressants	0.007	0.93
Melatonin *	1.986	0.16

* In the test, 2 cells had an expected cell frequency <5.

**Table 3:** Correlation between gender and the pre-existing conditions lipometabolic disorder, diabetes mellitus, cardiovascular disease, COPD, asthma bronchiale and acromegaly. Results of the chi-square tests. V: measure of the effect size.

	χ^2^ (1)	*p*	V
Lipometabolic disorder	4.62	0.03	0.20
Diabetes mellitus *	4.34	0.04	0.20
Cardiovascular disease	3.19	0.07	-
COPD *	0.72	0.40	-
Asthma bronchiale *	0.05	0.94	-
Acromegaly *	1.48	0.22	-

* In the test, 2 cells had an expected cell frequency <5.

## Data Availability

The data presented in this study are available on request from the corresponding author. The data are not publicly available due to privacy and ethical reasons.
